# Ovarian Non-Hodgkin Lymphoma Revealed by Bone Metastasis: A Rare Pediatric Case

**DOI:** 10.1155/2021/8826688

**Published:** 2021-04-20

**Authors:** M. Elbaz, S. Hiroual, F. Boukis, H. Raiss, A. Matrane, J. Elhoudzi

**Affiliations:** ^1^Pediatric Hematology and Oncology Department, Mohamed VI University Hospital, Caddi Ayyad University, Marrakesh, Morocco; ^2^Nuclear Medicine Department, Mohamed VI University Hospital, Caddi Ayyad University, Marrakesh, Morocco; ^3^Anatomo-pathology Department, Mohamed VI University Hospital, Caddi Ayyad University, Marrakesh, Morocco

## Abstract

Ovary involvement of non-Hodgkin lymphoma (NHL) is rare. We report a rare case of ovarian NHL during adolescence revealed by bone metastasis. The diagnosis of malignant lymphoma was established after adnexectomy and histological study of the excised tissue. The tumor was classified as a diffuse large B-cell lymphoma. The patient has been treated according to the LMB French protocol with good outcome after two years. Although NHL is a rare ovarian neoplasm, it is essential to establish an accurate diagnosis as early as possible for therapeutic purposes.

## 1. Introduction

Ovarian infiltration in pediatric non-Hodgkin lymphoma (NHL) is rare, and information on outcome is scarce and mainly based on case reports and small series. Ovarian involvement by NHL is usually secondary, occurring as a part of systemic disease. Localized presumably primary NHL of the ovary (PONHL) is rare, accounting for 0.5% of all NHL and 1.5% of all ovarian tumors [1].

We report a case of 12-year-old girl diagnosed with diffuse large B-cell lymphoma (DLBCL) revealed by bone metastasis with a good outcome.

## 2. Case Report

**Figure 1:**
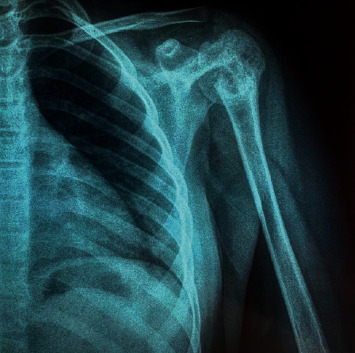
Plain film reveals osteolytic metastases in the left humerus.

**Figure 2:**
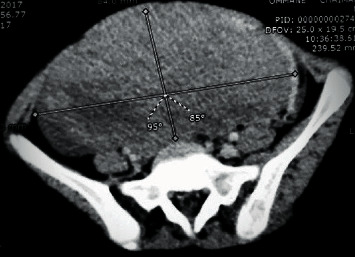
Contrast-enhanced CT shows a heterogeneous density abdomino-pelvic tumor.

**Figure 3:**
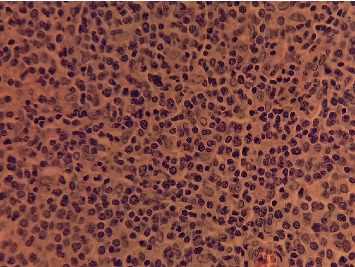
Cytology of pediatric ovarian lymphoma: diffuse large B-cell lymphoma (40x).

**Figure 4:**
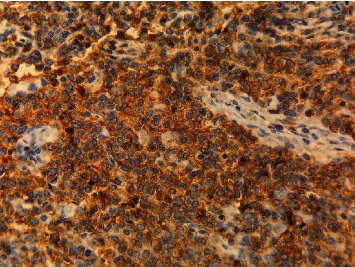
CD45 staining of the tumor cells (40x).

**Figure 5:**
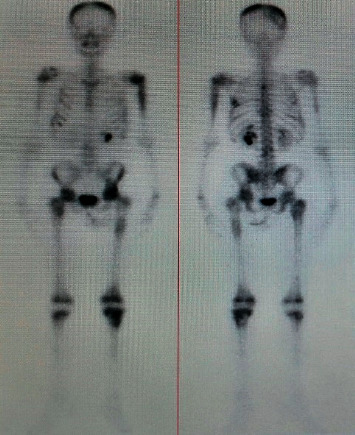
Skeletal scintigraphy showing multiple bone metastases.

A 12-year-old girl (no puberty) presented with localized pain of the left shoulder evolving for 9 months, and plain radiography done in the emergency showed osteolytic lesions without rupture of the cortex of the upper end of the left humerus (Figure 1) confirmed on computerized tomography (CT) scan. A bone biopsy done twice concluded the presence of rare atypical cramped cells. In the meantime, the patient had lost weight with an apparition of abdominal distension. On examination, she had pallor, a large mass was palpable per abdomen in the umbilical region measuring 15 × 9 cm, with firm consistency, and the lower limit of the mass was not palpable. There was no significant peripheral lymphadenopathy. Further clinical investigation showed elevated levels of serum lactate dehydrogenase (1250 IU/L), but serum was negative for alpha-fetoprotein and human chorionic gonadotrophin. Ultrasonography revealed a solid mass in the pelvic cavity. Abdominal CT scan revealed a 10-cm diameter abdomino-pelvic mass of heterogeneous density without calcification (Figure 2), whose origin is difficult to assess. A laparotomy was performed; a tumor of the left ovary with adhesion to the omentum was identified. The uterus and the right ovary were normal. Subsequently, a left salpingo-oophorectomy was performed. On pathological investigation, the tumor tissue was composed of mild-to-large, round-to-polygonal atypical lymph cells with diffuse but densely packed chromatin. Immunohistochemistry indicated that the cells were positive for CD20 and CD45 and Ki67 (about 80%) but negative for CD3, BCL2, and BCL6 (Figures 3 and 4). The diagnosis made was a diffuse large B-cell lymphoma (DLBCL) of the left ovary with bone metastasis confirmed by bone scintigraphy which revealed multiple lesions with intensive radioactive accumulation in the left humerus, point of the left scapula, upper third of the left femur, the skull, and the second dorsal vertebra (Figure 5). The bone marrow aspiration and cerebral fluid cytology findings were normal. The patient was treated according to the French LMB protocol, started with a cytoreductive COP regimen (low dose of cyclophosphamide), followed by two induction COPADM cycles (high dose of methotrexate, cyclophosphamide, vincristine, doxorubicin, and prednisone), followed by 2 cycles of CYM (high dose of methotrexate and cytarabine). The patient is currently seen in consultation with a decline of two years, and she has reached puberty.

## 3. Discussion

Lymphomas of the ovary may occur at any age, but mostly women in their 40s are affected [2]. Pediatric cases of ovarian NHL are rare, which is similar to what has been reported in adults [3].

Ovarian involvement by lymphoma can be primary or secondary. The secondary involvement is either as an early metastasis in occult extraovarian neoplasms or as a generalized metastatic disease [4]. Primary ovarian lymphoma (POL) is a very rare entity in the ovary due to absence of lymphoid tissue within the ovary. A number of theories looked at the pathogenesis and the origin of POL, and some of them suggested that it originates from lymphocytes surrounding blood vessels at the hilum and related to the corpus luteum. Most authors consider PONHL as a local involvement of a systemic disease [5].

The consensus surrounding whether some ovarian lymphomas can be considered truly primary and not merely a localized initial manifestation of a generalized disease remains controversial [4]. Fox et al. proposed the following criteria for the diagnosis of PONHL [6]: (1) at the time of diagnosis, the lymphoma is clinically confined to the ovary and a complete investigation fails to reveal evidence of lymphoma elsewhere. However, an ovarian lymphoma can still be considered as primary if it has spread to immediately adjacent lymph nodes or if it has directly spread to infiltrate immediately adjacent structures. (2) The peripheral blood and bone marrow should not contain any abnormal cells. (3) If further lymphomatous lesions occur at sites remote from the ovary, then at least several months should have elapsed between the appearance of the ovarian and extraovarian lesions. The bone metastasis in our case may argue against a primary ovarian disease.

Most pediatric cases showed a B-cell NHL immunophenotype, of which the majority had a Burkitt type, which may be a reflection of the most common lymphoma found in children [7, 8]. In reported cases of adult ovarian NHL, DLBCL is found, followed by Burkitt lymphoma [3, 4]. Nevertheless, it may also stress the specific tendency of homing of malignant B-cell NHL cells rather than T-NHL cells to infiltrate ovarian tissue. In our case, it was DLBCL of the ovary, and it appears to be the most common type of primary ovarian NHL [2], but as our knowledge, no cases of pediatric diffuse large B-cell lymphoma of the ovary with bone metastasis were published before.

Specific clinical characteristics are difficult to describe. All pediatric cases presented with abdominal pain and/or abdominal mass [8, 9], which again is comparable with the presentation as described in adult cases [4, 10]. Our case has the particularity to be revealed initially by bone pain secondary to bone metastasis.

Chemotherapy regimens are considered the most appropriate therapeutic option for patients with NHL [1]. Surgical treatment is not the treatment of choice in patients with ovarian lymphoma. However, surgical intervention plays an important role in the diagnostic process providing clinical information, staging, and immunohistological examination. Fine needle biopsy can avoid surgery in some cases if the diagnosis of lymphoma is considered [11]. If surgical therapy is needed, it should be conservative to preserve fertility, especially in bilateral forms [12].

Prognosis of ovarian lymphomas is often poorer than nodal lymphomas due to inaccurate or delayed diagnosis [13]. POL has a better survival rate probably due to the early presentation and early stage at diagnosis. A 5-year survival rate in POL is 80%, while secondary cases are only 33% [14]. The declines in this case are good at 2 years of follow-up.

## 4. Conclusion

This article presents a unique case of ovarian DLBCL with bone metastasis in a 12-year-old girl with good outcome. Unfortunately, ovarian malignancies during childhood and adolescence present several difficulties, mostly caused by late diagnosis. The best treatment option seems to be chemotherapy. Physicians should be aware of this rare presentation to avoid radical surgery, which is unnecessary.

## References

[1] Yamada T., Mori H. (2004). Ovarian metastases of lymphomas and other hematological malignancies. *Journal of Gynecologic Oncology*.

[2] Vang R., Medeiros L. J., Warnke R. A., Higgins J. P., Deavers M. T. (2001). Ovarian non-hodgkin’s lymphoma: a clinicopathologic study of eight primary cases. *Modern Pathology*.

[3] Dimopoulos M. A., Daliani D., Pugh W., Gershenson D., Cabanillas F., Sarris A. H. (1997). Primary ovarian non-hodgkin’s lymphoma: outcome after treatment with combination chemotherapy. *Gynecologic Oncology*.

[4] Monterroso V., Jaffe E. S., Merino M. J., Medeiros L. J. (1993). Malignant lymphomas involving the ovary. *The American Journal of Surgical Pathology*.

[5] Crawshaw J., Sohaib S. A., Wotherspoon A., Shepherd J. H. (2007). Primary non-Hodgkin’s lymphoma of the ovaries: imaging findings. *The British Journal of Radiology*.

[6] Fox H., Langley F. A., Govan A. D. T., Hill A. S., Bennett M. H. (1988). Malignant lymphoma presenting as an ovarian tumour: a clinicopathological analysis of 34 cases. *BJOG: An International Journal of Obstetrics and Gynaecology*.

[7] Patte C., Auperin A., Michon J. (2001). The Societe Francaise d’Oncologie Pediatrique LMB89 protocol: highly effective multiagent chemotherapy tailored to the tumor burden and initial response in 561 unselected children with B-cell lymphomas and L3 leukemia. *Blood*.

[8] Van Dorp W., Owusuaa C., Laven J. S. E., van den Heuvel-Eibrink M. M., Beishuizen A. (2013). Characteristics and outcome of pediatric non-Hodgkin lymphoma patients with ovarian infiltration at presentation. *Pediatric Blood & Cancer*.

[9] Creatsas G. K., Hassan E. A., Deligeoroglou E. K., Markaki S. G., Michalas S. P. (1997). Non-Hodgkin’s ovarian lymphoma during adolescence: report of two cases. *Journal of Pediatric and Adolescent Gynecology*.

[10] Vang R., Medeiros L. J., Fuller G. N., Sarris A. H., Deavers M. (2001). Non-Hodgkin’s lymphoma involving the gynecologic tract: a review of 88 cases. *Advances in Anatomic Pathology*.

[11] Yadav R., Balasundaram P., Mridha A. R., Iyer V. K., Mathur S. R. (2016). Primary ovarian non-Hodgkin lymphoma: diagnosis of two cases on fine needle aspiration cytology. *Cytojournal*.

[12] Türken A., Ciftci A. O., Akçören Z., Köseoglu V., Akata D., Şenocak M. E. (2000). Primary ovarian lymphoma in an infant: report of a case. *Surgery Today*.

[13] Trenhaile T. R., Killackey M. A. (2001). Primary pelvic non-hodgkin’s lymphoma. *Obstetrics & Gynecology*.

[14] Crasta J., Vallikad E. (2009). Ovarian lymphoma. *Indian Journal of Medical and Paediatric Oncology*.

